# Redistribution of organ specific blood flow in response to food ingestion measured by R-R-interval averaged golden-angle spiral phase contrast MRI

**DOI:** 10.1186/1532-429X-18-S1-O92

**Published:** 2016-01-27

**Authors:** Jakob A Hauser, Vivek Muthurangu, Andrew Taylor, Jennifer A Steeden, Alexander Jones

**Affiliations:** grid.83440.3b0000000121901201Institute of Cardiovascular Science, University College London, London, UK

## Background

The ingestion of food is known to increase mesenteric blood flow significantly. However, it remains unknown whether this increased flow demand to the intestine is compensated by an increase in total cardiac output (CO) alone, or by redistribution of blood flow from other organs, caused by changes in organ-specific vascular resistance. Non-invasive studies on the vascular response of small arteries have been scarce due to technical constraints, as conventional as well as MRI-based methods for non-invasive flow assessment are time-consuming and observer-dependent. We present a novel, comprehensive phenotyping model of the hemodynamic changes caused by the ingestion of a high-energy liquid meal, assessed by novel MRI sequences for the rapid assessment of small vessel blood flow.

## Methods

After a 12-hour fasting period, 20 healthy volunteers (7 female; mean age 32.2 ± 8.5 years, range 18-52) underwent MRI assessment of ventricular volumes, segmental aortic and organ specific blood flow. After ingestion of a high-energy liquid meal, image and flow acquisitions were repeated every 10 minutes for 1 hour. Radial kt-SENSE imaging was performed for rapid measurement of ventricular volumes during free breathing. Small vessel flow to the brain, the celiac artery, the superior mesenteric artery (SMA), the kidneys and the legs was quantified by R-R-interval averaged golden-angle spiral phase contrast MRI. Blood pressure was measured in 5-minute intervals to calculate vascular resistance through division by blood flow. Changes over time were assessed using multilevel linear regression and corrected for sex, body mass index and age.

## Results

Ingestion of the meal was associated with a substantial drop in systemic vascular resistance and a significant increase in total CO by an average 1.1 L/min, caused by a rise in, both, stroke volume and heart rate. Mean arterial blood pressure remained stable. Vascular resistance in the SMA decreased substantially within 10 minutes and reached a minimum after 50 minutes, resulting in a more than 4-fold increase of intestinal blood flow (0.3 to 1.3 L/min). There was a moderate but significant decrease in renal vascular resistance. No changes in the vascular resistance of the brain, celiac artery or the limbs were observed. End-diastolic volume remained unaffected, however, left ventricular ejection fraction increased significantly.

## Conclusions

In conclusion, this is the first study to characterize systemic and regional changes in blood flow after ingestion of a high-energy liquid meal in humans using a novel dynamic MRI approach. Our findings show that the increase in CO output seen after ingestion of a meal is the effect of an increase of total CO alone, without any significant redistribution from non-digestive organ systems.

**Figure 1:**
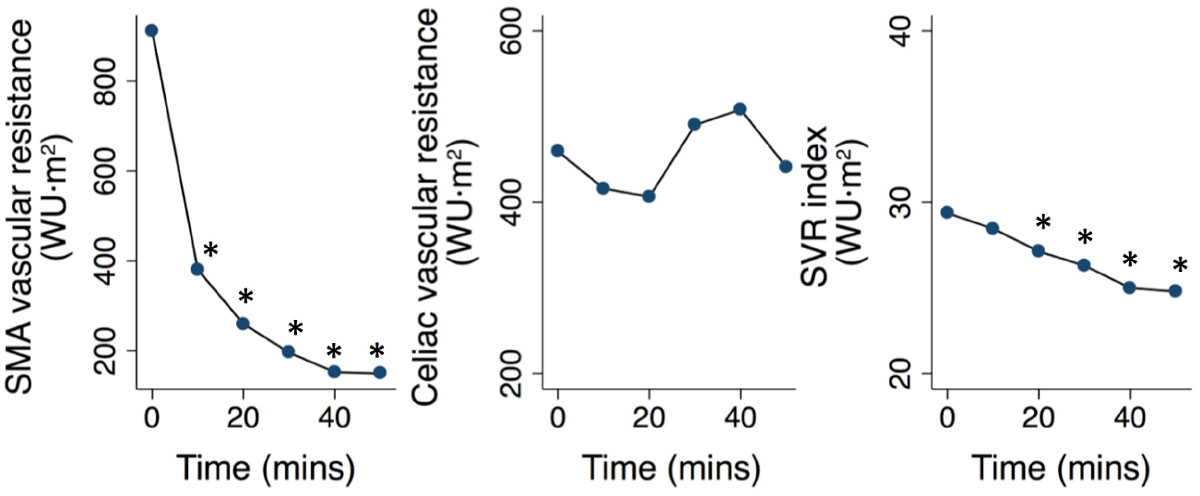
**Changes in mesenteric (SMA), celiac and systemic vascular resistance (SVR)**. * p < 0.001

**Figure 2:**
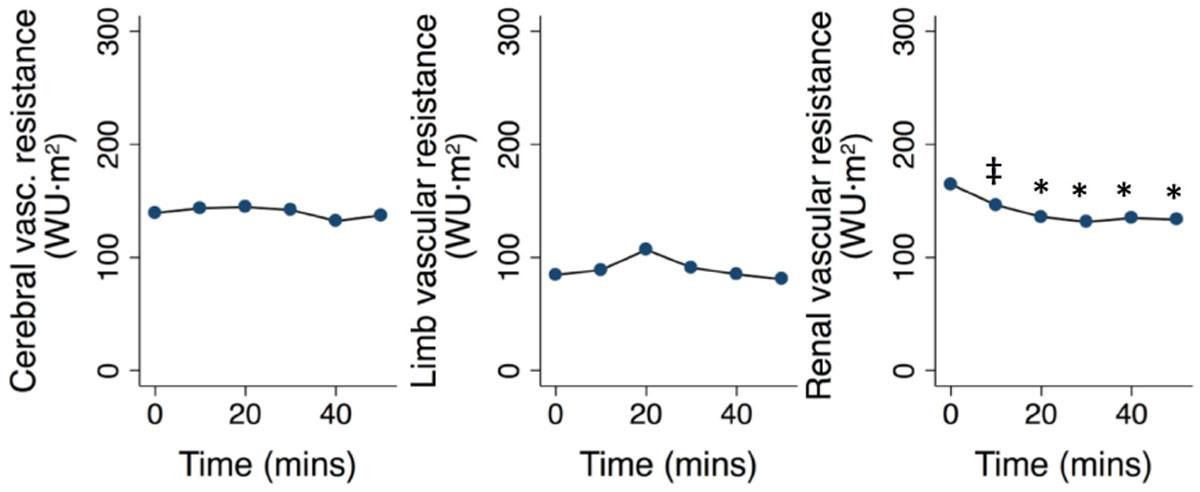
**Changes in cerebral, limb and renal vascular resistance**. ‡ p < 0.05; * p < 0.001

